# IL-1β Suppresses Innate IL-25 and IL-33 Production and Maintains Helminth Chronicity

**DOI:** 10.1371/journal.ppat.1003531

**Published:** 2013-08-01

**Authors:** Mario M. Zaiss, Kendle M. Maslowski, Ilaria Mosconi, Nadine Guenat, Benjamin J. Marsland, Nicola L. Harris

**Affiliations:** 1 Global Health Institute, École Polytechnique Fédèrale de Lausanne (EPFL), Lausanne, Switzerland; 2 Department of Biochemistry, University of Lausanne, Lausanne, Switzerland; 3 Department of Pneumology, Centre Hospitalier Universitaire Vaudois (CHUV), Lausanne, Switzerland; MRC National Institute for Medical Research, United Kingdom

## Abstract

Approximately 2 billion people currently suffer from intestinal helminth infections, which are typically chronic in nature and result in growth retardation, vitamin A deficiency, anemia and poor cognitive function. Such chronicity results from co-evolution between helminths and their mammalian hosts; however, the molecular mechanisms by which these organisms avert immune rejection are not clear. We have found that the natural murine helminth, *Heligmosomoides polygyrus bakeri* (Hp) elicits the secretion of IL-1β *in vivo* and *in vitro* and that this cytokine is critical for shaping a mucosal environment suited to helminth chronicity. Indeed in mice deficient for IL-1β (IL-1β^−/−^), or treated with the soluble IL-1βR antagonist, Anakinra, helminth infection results in enhanced type 2 immunity and accelerated parasite expulsion. IL-1β acts to decrease production of IL-25 and IL-33 at early time points following infection and parasite rejection was determined to require IL-25. Taken together, these data indicate that Hp promotes the release of host-derived IL-1β that suppresses the release of innate cytokines, resulting in suboptimal type 2 immunity and allowing pathogen chronicity.

## Author Summary

Parasitic soil-transmitted helminths (STH) live, feed and mate within the intestine of their mammalian hosts. Infection is caused by ingestion of eggs and larvae or by active penetration of the skin by larvae from contaminated soil in poverty stricken areas lacking adequate sanitation. Infection with soil-transmitted helminths results in a wide range of symptoms including intestinal complications (diarrhea, abdominal pain), anemia, general malaise and nutritional deficiencies that negatively impact on working and learning capacities and impair physical growth. The host typically raises a strong type 2 immune response against the invading helminth; however, the majority of helminthes have developed the capacity to escape effective immunity and can remain chronically within the intestine for years. We explored the mechanisms responsible for helminth chronicity using a murine model of *Heligmosomoides polygyrus bakeri* (Hp) infection. We found that Hp is able to induce IL-1β secretion in the intestine and that this cytokine acts to suppress the production of the innate cytokines IL-25 and IL-33. Furthermore, we show that parasite rejection requires IL-25 indicating that suboptimal production of this cytokine in the presence of IL-1β is responsible for the ability of IL-1β to promote parasite chronicity. Our study provides new insights into the mechanisms used by helminths to establish chronic infections, and reveals a novel role for IL-1β in protective immunity.

## Introduction

Soil-transmitted helminths (STH) are parasitic nematodes that currently infect billions of people worldwide [1]. They can live for many years as adult worms within the human gastrointestinal tract, with the heaviest worm burdens found in preschool and school-age children living in impoverished communities [2]. *Heligmosomoides polygyrus bakeri* (Hp) is a widely used murine STH that mimics the life-cycle and chronicity of many human helminths. It is a natural parasite of mice that enters the gastro-intestinal tract as third-stage infective larvae (L3), then penetrates the epithelial cell barrier of the small intestine to mature within the submucosa to an L4 stage, during which period it elicits a type 2 dominated inflammatory response [3], [4]. The parasite eventually exits the intestinal mucosa to populate the intestinal lumen where it establishes a chronic infection as a sexually mature adult [5]. Although the mechanisms by which Hp establishes chronicity in its host remain unclear, it is well established that this helminth possesses potent immunomodulatory properties. Indeed Hp has been reported to ameloriate various inflammatory diseases including allergic asthma [6], [7] and inflammatory bowel disease [7], [8], to directly modulate dendritic cell (DC) function [9] and to promote de novo Foxp3 expression by splenocytes *in vitro* [10]. Protective immunity is thought to be mediated largely against the tissue invasive L4 stage [11], [12], and immune damage inflicted on the parasite during this stage can lead to a halt in the life cycle [11], [12] or the emergence of damaged worms that are more easily expelled from the intestinal lumen [13].

The IL-1 cytokine family comprises 11 members, including IL-1β. Production of active IL-1β is a tightly controlled process. Pro-inflammatory stimuli can activate the expression of the proform of IL-1β, while maturation is regulated by inflammasome formation. Inflammasomes, such as the Nlrp3 and Nlrp6 inflammasomes, are molecular platforms comprised of a NOD-like receptor (NLR) family protein, the adaptor protein apoptosis-associated speck-like protein containing a caspase recruitment domain (ASC) and pro-caspase-1. Upon stimulation these proteins oligomerize and enable auto-activation of caspase-1 (Casp1), which can then mediate cleavage, and activation, of IL-1β [14]. Among the inflammasomes described to date, the Nlrp3 inflammasome is the best characterized. The Nlrp3 inflammasome is known to be activated by a wide range of stimuli such as asbestos, silica, monosodium urate crystals (MSU), adenosine triphosphate (ATP) and ultraviolet B (UVB) irradiation [15], [16]. So far IL-1β production has been linked to several pathological diseases including rheumathoid arthitis [17], gout [18] and type 2 diabetes [19], and it was shown that treatment with Anakinra, an IL-1β receptor antagonist, improved disease outcomes [20], [21], [22]. IL-1β is also upregulated in the intestines of patients suffering from inflammatory bowel disease [23], [24], [25], [26]. Pathogens can elicit IL-1β secretion and it is necessary for the effective clearance of *Salmonella typhimurium* (Nlrp3 and Nlrc4 inflammasomes) [27], *Shigella flexneri* (Nlrc4 inflammasome) [28], and *Legionella pneumophila* [29]. Lastly, a recent publication indicated that eggs from the cestode helminth, *Schistosoma mansoni*, can elicit IL-1β secretion in an Nlrp3 inflammasome-dependent manner [30].

In the current report we uncover a novel role for IL-1β production in promoting the chronicity of intestinal helminth infection. Hp infection elicited a strong early production of IL-1β, which was determined to favour the development of parasite chronicity through its ability to suppress helminth-induced IL-25, resulting in attenuated type 2 immunity. These findings uncover one of the means by which Hp establishes a chronic infection within its murine host, and represents the first evidence of a negative role for IL-1β in protective immunity following pathogen infection.

## Results

### Hp parasites and their excretory/secretory products activate the Nlrp3 inflammasome to elicit IL-1β release

**Figure 1:**
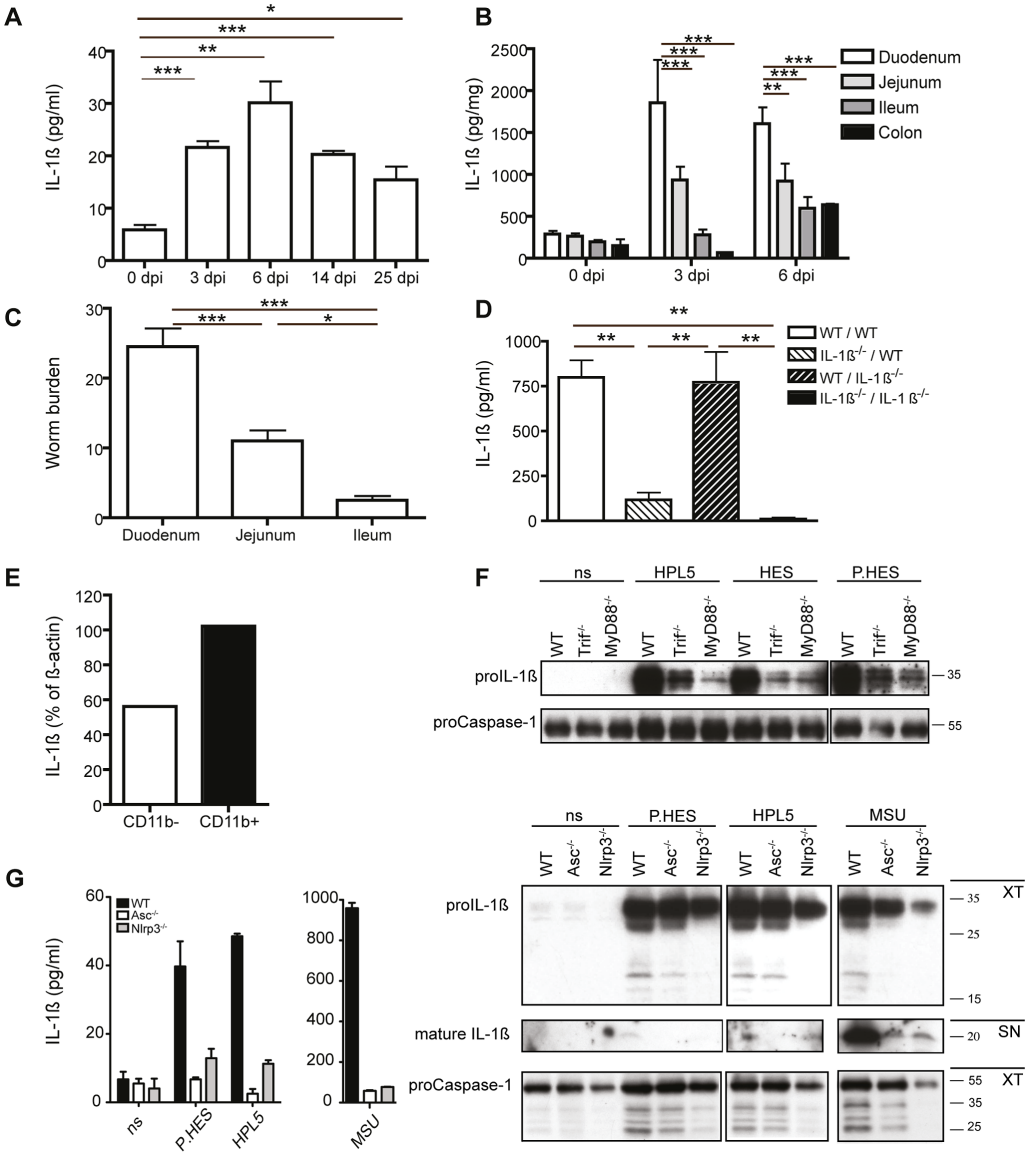
Hp infection elicits IL-1β secretion. (**A–E**) Mice were administered 200 L3 Hp by oral gavage. IL-1β protein levels were measured by ELISA in (**A**) the peritoneal wash and (**B**) intestinal tissue culture supernatants at the indicated timepoints. (**C**) Hp worm counts were performed for defined segments of the small intestine from WT (C57BL/6) at 13 dpi. (**D**) IL-1β was measured by ELISA for duodenum tissue culture supernatants of day 6 infected bone marrow chimera mice (donor strain/recipient strain). Results are representative of at least 3 independent experiments (n = 5 per group) and expressed as mean ± SEM. (**E**) Intestinal lamina propria CD11b^+^ and CD11b^−^ cells were isolated at 6 dpi and analyzed by Western blot for IL-1β expression. Band intensity for IL-1β and β-actin was determined using Adobe Photoshop CS3. The ratio between IL-1β and the control protein β-actin band intensity was then determined. IL-1β is expressed as percent of β-actin intensity. (**F**) BMMs from WT (C57BL/6), Trif*^−/−^* or MyD88*^−/−^* mice were stimulated *in vitro* with HPL5 (100 µg/mL), HES (5 µg/mL) or P.HES (5 µg/mL) for 4 hours and cell extracts were analyzed for pro-IL-1β expression by Western blot. (**G**) BMMs from WT (C57BL/6), Asc*^−/−^* or Nlrp*3^−/−^* mice were stimulated *in vitro* with P.HES, HPL5 or LPS plus MSU and culture supernatants (SN) were analyzed for active IL-1β by ELISA. Cell extracts (XT) and SN were also analyzed for pro- or mature- IL-1β by Western blot. Pro-Casp1 was used as a control protein. XT blots for IL-1β and Casp1 demonstrate the pro-forms, and the observed lower bands in the IL-1β and Casp1 blots are cleavage products. Error bars represent means of triplicate cultures ± SEM and the experiment was repeated 3 times.

We analyzed factors induced early after infection of WT (C57BL/6) mice with Hp and observed a significant increase in IL-1β cytokine levels in the peritoneal wash (Fig. 1A), a site previously shown to contain inflammatory cells following Hp infection [31]. IL-1β was also elevated in the intestine (Fig. 1B) with a peak at 3–6 days post infection (dpi). Here, IL-1β production was most prominent in the duodenum, and to a lesser extent within the jejunum, correlating with the sites of heaviest worm burdens (Fig. 1C). We next generated bone marrow (BM) chimeras using WT and IL-1β^−/−^ mice to identify the cellular compartment responsible for IL-1β secretion. Fig. 1D shows similar increases in IL-1β cytokine levels from duodenum tissue cultures at 6 dpi in IL-1β^−/−^ recipient mice reconstituted with WT BM cells (IL-1β^−/−^/WT) and WT (WT/WT) controls, whilst very little IL-1β was detected in WT recipients reconstituted with IL-1β^−/−^ BM cells. Western blot analysis of hematopoietic cells isolated from the intestinal lamina propria tissue layer of Hp infected WT mice at 6 dpi demonstrated that both CD11b^+^ and CD11b^−^ cells can express IL-1β, indicating that multiple cells types are likely to contribute to production of this cytokine (Fig. 1E). However, predominantly more IL-1β expression on a per-cell-basis was noted within the CD11b^+^ compartment, indicating that CD11b^+^ macrophages may be a particularly rich source of this cytokine following helminth infection (Fig. 1E).

IL-1β plays a key role during many inflammatory responses [32]. Inflammatory stimuli can induce expression of proIL-1β through NFκB activation, but this requires cleavage and release of the mature form to be active. Cleavage of proIL-1β can occur via inflammasome activation of caspase-1. To determine whether Hp could induce expression of proIL-1β we stimulated WT, Trif^−/−^ and MyD88^−/−^ BM derived macrophages (BMM) with whole extracts from L5 Hp worms (HPL5), L5 Hp excretory secretory proteins (HES)(which contains low levels of contaminating LPS) or pyrogen-free HES (P.HES). We then examined proIL-1β expression by Western blot. WT BMMs showed strong expression of proIL-1β following stimulation with HPL5, HES or P.HES after 4 hours (Fig. 1F). Trif^−/−^ and, even more dramatically MyD88^−/−^ BMMs, showed reduced levels of proIL-1β following HPL5, HES or P.HES stimulation (Fig. 1F), demonstrating that Hp products can activate NFκB pathways dependent on both Trif and MyD88. Next, to determine whether Hp products could activate inflammasome activity and mature IL-1β secretion we stimulated WT, Asc^−/−^ or Nlrp3^−/−^ BMMs with HPL5, P.HES or LPS plus MSU (as a positive control). After 18 hours IL-1β expression was detected by Western blot and ELISA. As expected LPS plus MSU treatment induced inflammasome activation in WT BMMs, which was absent in Asc^−/−^ and Nlrp3^−/−^ BMMs (Fig. 1G). WT, Asc^−/−^ and Nlrp*3* ^−/−^ BMMs all showed expression of proIL-1β following stimulation with the Hp products, demonstrating that the cells are equally viable and that proIL-1β expression is not dependent on Asc or Nlrp3 (Fig. 1G). WT, but not Asc^−/−^ or Nlrp3^−/−^, BMMs demonstrated cleavage and secretion of IL-1β (Fig. 1G), indicating that Hp products can activate the NLRP3 inflammasome. Similar results were obtained using THP-1 cells with knock-down (kd) for ASC or NLRP3. THP-1 cells with mock shRNA treatment released IL-1β when stimulated with HPL5, HES or P.HES, whilst ASCkd or NLRP3kd THP-1 cells did not (Fig. S1). No differences in IL-1β release were detected for BMM or THP-1 cells stimulated with HES or P.HES, suggesting that contaminating LPS did not contribute to IL-1β secretion, but that it resulted from direct stimulation by helminth-derived products. Taken together, these data indicate that Hp products can stimulate IL-1β secretion by hematopoietic cells through a pathway involving the Nlrp3 inflammasome.

### IL-1β promotes chronic Hp infection in mice

**Figure 2:**
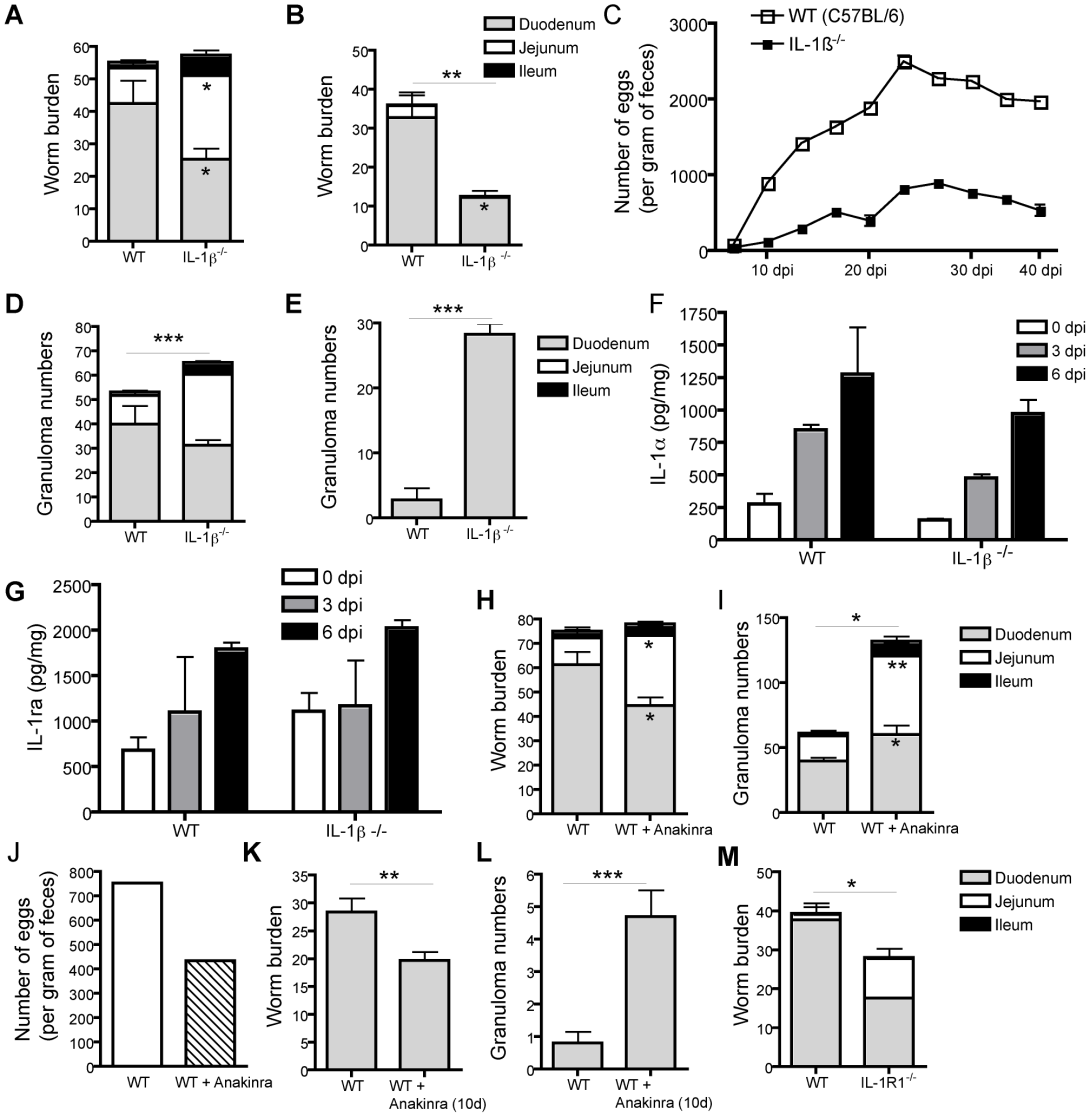
IL-1β promotes chronicity of Hp infection in mice. Mice were administered 200 L3 Hp by oral gavage (**A–M**). The number of adult worms were determined for the entire and defined segments, of the small intestine of WT (C57BL/6) and IL-1β^−/−^ mice at (**A**) 13 dpi and (**B**) 40 dpi. (**C**) Fecal egg counts were determined for WT (C57BL/6) and IL-1β^−/−^ mice throughout the course of the experiment (data represents pooled feces of 2–3 individual cages per strain, n = 2–5 mice per cage). Numbers of type 2 granulomas were determined for the entire and defined segments of the small intestine of WT (C57BL/6) and IL-1β^−/−^ mice at (**D**) 13 dpi and (**E**) 40 dpi. IL-1α (**F**) and IL-1ra (**G**) protein levels were measured by ELISA in duodenum tissue culture supernatants at the indicated timepoints after Hp infection. (**H–I**) WT (C57BL/6) mice were administered 200 L3 Hp by oral gavage and additionally received 100 µl of PBS (WT) or PBS plus 50 mg/kg Anakinra (WT+Anakinra) via i.p. injection every day from 0–10 dpi. The number of (**H**) adult Hp worms and (**I**) type 2 granulomas were determined for the entire and defined segments of the small intestine at 10 dpi. (**J**) Fecal egg counts were determined at 10 dpi (data represents pooled feces from 1 cage per strain, n = 5 mice per cage). (**K–L**) WT (C57BL/6) mice were administered 200 L3 Hp larvae by oral gavage and additionally received 100 µl of PBS (WT) or PBS plus 50 mg/kg Anakinra (WT+Anakinra (10 d)) via i.p. injection every day from 0–10 dpi. Mice were sacrificed at day 36 dpi and the number of (**K**) adult worms and (**L**) type 2 granulomas determined for the entire and defined segments of the small intestine. (**M**) Number of adult worms were determined for the entire and defined segments of the small intestine of WT (C57BL/6) and IL-1R1^−/−^ mice at 13 dpi. All data are representative of 3 independent experiments (n = 5–10 per group) and expressed as mean ± SEM.

We next investigated the functional role of IL-1β production during Hp infection. IL-1β^−/−^ mice infected with Hp demonstrated similar worm burdens at early timepoints when mature L5 Hp first enter the intestinal lumen (Fig. 2A). However, whilst the bulk of worms in WT mice were located in the duodenum, IL-1β^−/−^ mice harbored increased numbers of worms in both the duodenum and jejunum (Fig. 2A). Location of adult worms further along the small intestine is typically associated with immune damage leading to impaired worm health, fecundity and more rapid expulsion [33]. In keeping with this, a strong reduction in Hp worms was noted along the entire small intestine of IL-1β^−/−^ mice at a late timepoint post-infection (day 40) (Fig. 2B). In addition, IL-1β^−/−^ mice exhibited reduced egg output over the entire timecourse of the experiment (Fig. 2C). Type 2 granulomas form around the invading larvae and resolve slowly after the larvae emerge as adults into the intestine. Thus large numbers of granulomas are typically seen within the first few weeks postinfection and decrease thereafter. IL-1β^−/−^ mice exhibited increased numbers of intestinal granulomas at both early and late timepoints following infection (Figs. 2D–E). We also observed an increased proportion of granulomas in the jejunum of IL-1β^−/−^ mice suggesting that Hp larvae are forced to invade the intestine at a more distal location (Fig. 2D). These data may indicate a very early increase in immune responsiveness in the absence of IL-1β. To ensure that the observed phenotype resulted from the absence of IL-1β, and not alterations to other components of the IL-1 signaling pathway, we measured IL-1α and IL-1ra cytokine levels in the intestinal duodenal tissue cultures of WT and IL-1β^−/−^ mice. These analyses showed that Hp infection increases both IL-1α and IL-1ra, however no significant differences were observed between WT and IL-1β^−/−^ mice (Figs. 2F–G).

### Anakinra treatment mimics the phenotype of IL-1β^−/−^ mice following Hp infection

To exclude any intrinsic defects in genetically targeted IL-1β^−/−^ mice, we also blocked IL-1β activity pharmacologically by treating WT (C57BL/6) mice daily with Anakinra. Similar to the results observed in IL-1β^−/−^ mice, Anakinra-treated WT mice did not show any difference in total worm burdens in the complete small intestine at early timepoints following infection, however an increased number of worms were located towards the posterior end of the small intestine indicating reduced parasite fitness (Fig. 2H). Anakinra treated mice also exhibited increased numbers of granulomas (Fig. 2I) and reduced egg outputs (Fig. 2J). In keeping with published data that immune damage to tissue- invasive larvae during the early phase of the immune response correlates with greater expulsion of adult worms, we could show that limiting Anakinra treatment to the first 10 days of infection was sufficient to decrease worm burdens (Fig. 2K) and increase granuloma numbers (Fig. 2L) during the chronic phase of infection (day 36 post-infection). IL-1R1^−/−^ also exhibited reduced worm burdens as compared to WT mice (Fig. 2M). Taken together, these data show that IL-1β normally acts to promote Hp chronicity.

### IL-1β attenuates Th2 cell immunity following Hp infection

**Figure 3:**
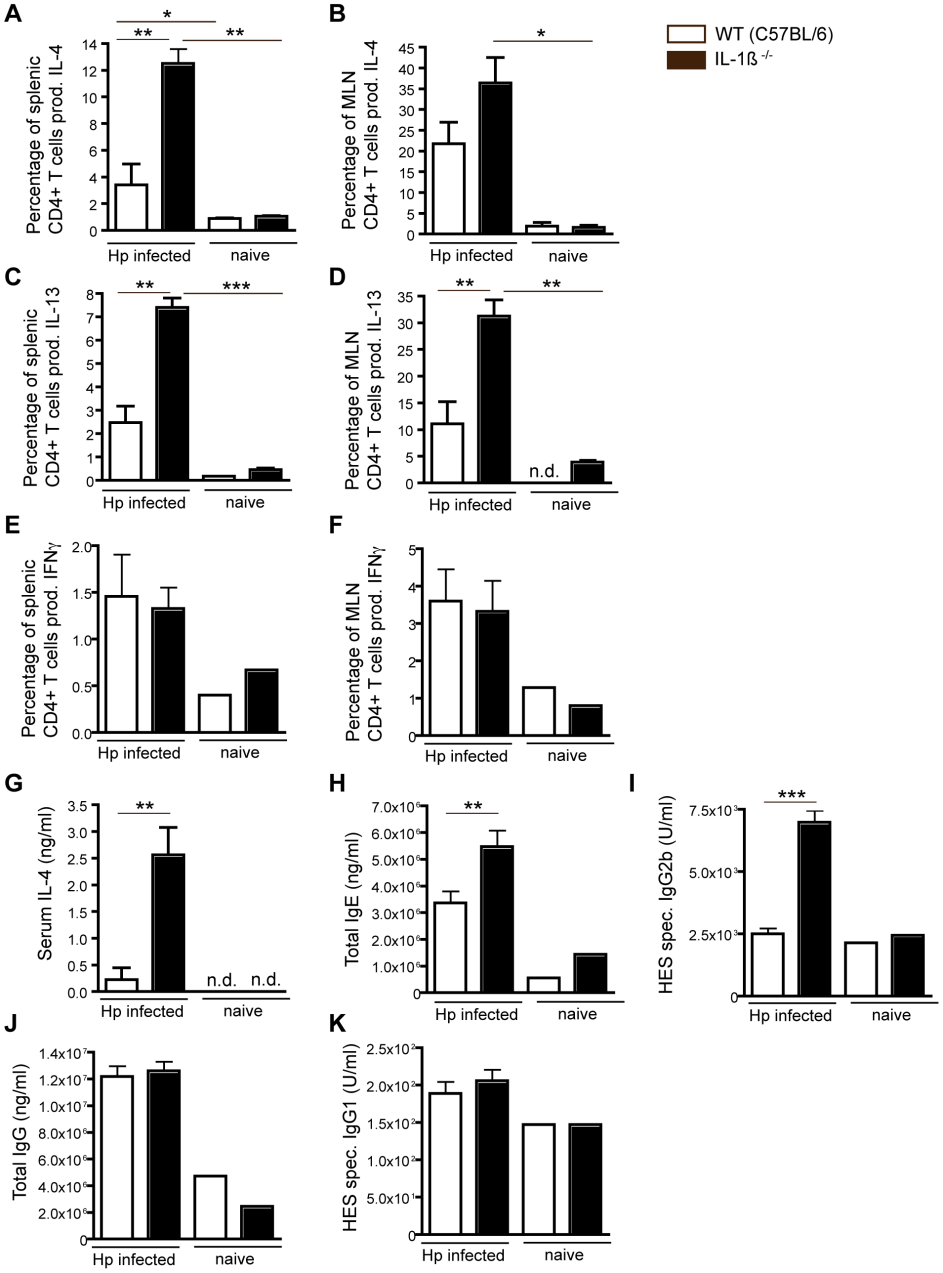
IL-1β negatively regulates the development of CD4^+^ Th2 cells following Hp infection. Mice were administered 200 L3 Hp by oral gavage. At 13 dpi single cell suspensions were made from the (**A, C, E**) spleen and (**B, D, F**) MLN, and cells restimulated with HES as described in the Materials and Methods. The percentage of CD4^+^ T cells secreting (**A, B**) IL-4, (**C, D**) IL-13 and (**E, F**) IFNγ is shown for WT (C57BL/6) or IL-1β^−/−^ mice. Serum was taken at 13 dpi and levels of (**G**) IL-4, (**H**) total IgE, (**I**) HES specific IgG2b, (**J**) total IgG and (**K**) HES specific IgG1 determined. All results are representative of 3 independent experiments (n = 5–7 per group) and expressed as mean ± SEM.

Protective immunity following challenge with Hp infection is known to require CD4^+^ T cells [34] and worm expulsion during primary infection is defective in SCID mice [35]. Moreover, IL-4 complex treatment can reduce worm numbers and fecundity in established Hp infection [35]. To determine whether IL-1β modulated Th2-type immunity we analyzed the peak CD4^+^ T cell response in IL-1β^−/−^ and WT mice following Hp infection. IL-1β^−/−^ mice showed a significantly increased percentage of IL-4 and IL-13 positive CD4^+^ T cells following *in vitro* restimulation of splenic or MLN cells with HES (Fig. 3A–D). By contrast no differences were found in the percentage of CD4^+^ cells secreting IFN-γ in response to HES stimulation (Figs. 3E–F). Cultures using cells from Hp infected mice cultured without HES, or cells from naive control mice cultured with HES, showed minimal numbers of cytokine positive cells (data not shown). Hp infection was not observed to result in the differentiation of IL-17 producing CD4^+^ cells in WT mice (Figs. S2A–B), nor could we detect HES-induced IL-17 production in the supernatants of cells obtained from Hp infected WT or IL-1β^−/−^ mice (data not shown). There were also no differences in the numbers of CD25^+^Foxp3^+^ Treg cells present in the MLN or spleen of infected WT versus IL-1β^−/−^ mice (Figs. S2C–D). In support of the increased Th2 immunity observed in IL-1β^−/−^ mice we also detected increased total serum IL-4 (Fig. 3G), serum IgE (Fig. 3H) and HES-specific IgG2b (Fig. 3I). Total IgG levels, which increase independently of IL-4 [12], didn't differ between WT and IL-1β^−/−^ mice (Fig. 3J). Only minimal increases in IgG1 were seen at this timepoint (Fig. 3K) and no HES-specific IgG2a could be detected (data not shown).

### IL-1β secretion negatively regulates Hp-induced IL-25 and IL-33 cytokine production

**Figure 4:**
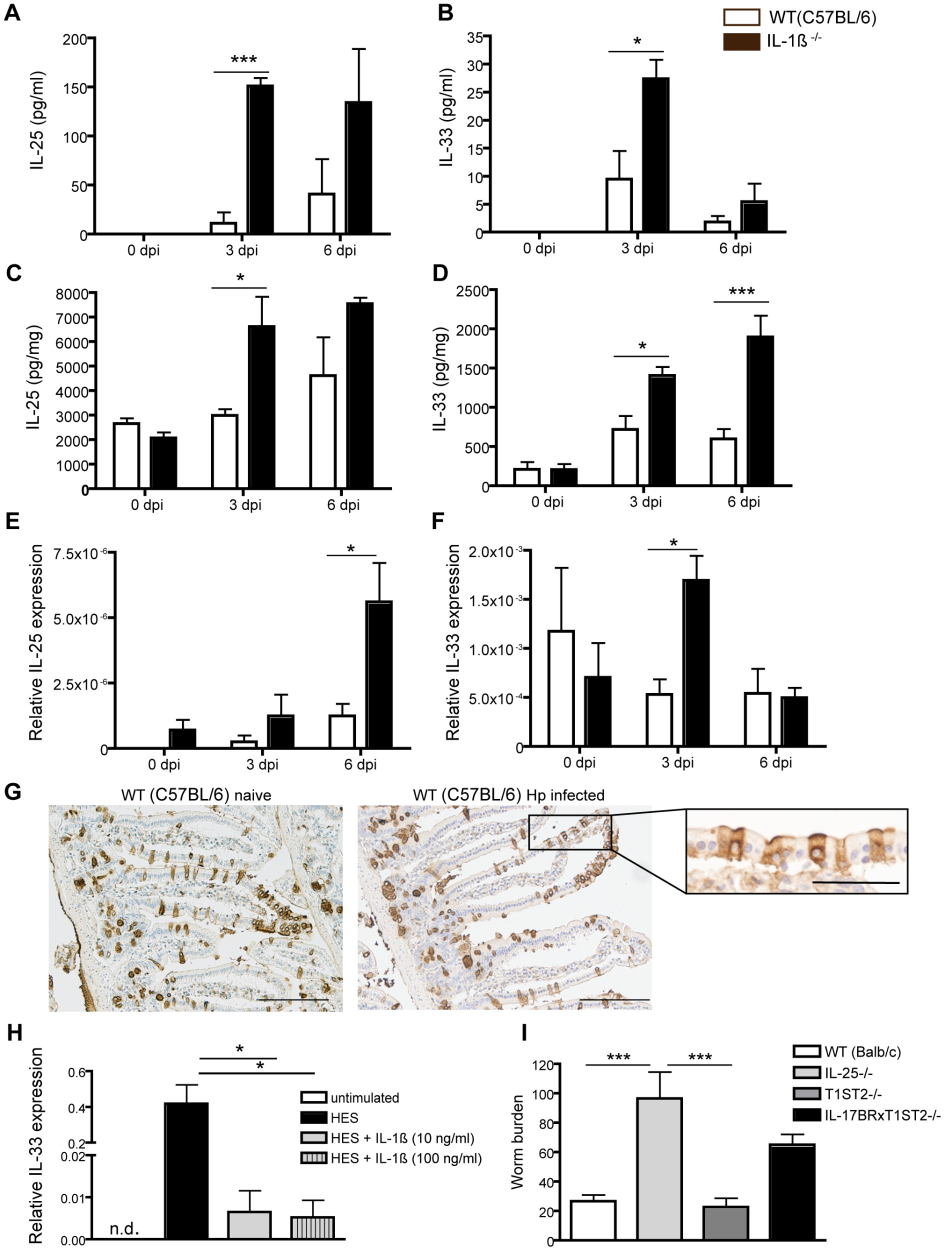
IL-1β attenuates Hp-induced IL-25 and IL-33 cytokine production. WT (C57BL/6), WT (Balb/c), IL-1β^−/−^ (C57BL/6), IL-25^−/−^(Balb/c), T1ST2^−/−^ (Balb/c) and IL-17Br×T1ST2^−/−^ (Balb/c) mice were administered 200 L3 Hp by oral gavage. (**A, C**) IL-25 and (**B, D**) IL-33 cytokine levels were measured by ELISA in (**A–B**) peritoneal wash and (**C–D**) intestinal duodenum tissue culture supernatants from at the indicated timepoints following Hp infection. mRNA expression for (**E**) IL-25 and (**F**) IL-33 in isolated intestinal epithelial cells were determined at 0, 3 and 6 dpi. (**G**) IL-1R1 expression was determined for the duodenum in naive and Hp infected WT (C57BL/6) at 6 dpi by immunohistochemistry. Scale bars represent 200 µm, and 50 µm for the inlet. (**H**) The mouse epithelial cell line (MSIE) was stimulated *in vitro* with HES (5 µg/ml) with or without additional rIL-1β and measured for IL-33 mRNA levels by RT-PCR. (**I**) Adult worm numbers determined for the entire small intestine at 45 dpi. All data are representative of 3 independent experiments (n = 5 per group), and expressed as mean ± SEM.

The cytokines IL-25 and IL-33 (a member of the IL-1 family) have been shown to play a crucial role in the regulation of type 2 cytokine production [36], [37], [38], to be produced rapidly following helminth infection, and to promote protective immunity against *Trichuris muris* [39], *Nippostrongylus brasilienis* [40] and Hp [41]. We therefore analyzed the impact of IL-1β on IL-25 and IL-33 production following Hp infection. Hp elicited the production of both IL-25 and IL-33, and this production was greatly enhanced in the absence of IL-1β (Figs. 4A–F). Increased IL-25 and IL-33 production in the absence of IL-1β could be detected in both the peritoneal wash (Figs. 4A–B) and intestinal duodenal tissue cultures (Figs. 4C–D). Anakinra treatment of WT mice also resulted in increased production of these cytokines, supporting our findings in IL-1β^−/−^ mice (Figs. S3A–B). In the intestine, Hp infection increased IL-25 and IL-33 mRNA expression was most predominant in isolated intestinal epithelial cells (IEC) (Figs. 4E–F), whilst it could not be detected in the lamina propria (data not shown). To determine the possible targets of IL-1β, we stained intestinal sections for expression of the IL-1R1. IL-1R1 was observed to be scattered throughout the intestine, and was particularly prodominant on IEC cells, although expression levels did not alter following infection (Fig. 4G). We therefore determined whether IL-1β could act directly on IECs to limit IL-25 and/or IL-33 production by stimulating IECs from the mouse small intestine (MSIE cells) [42] with HES in the presence or absence of rIL-1β. HES elicited the production of IL-33 mRNA by MSIE cells and this was attenuated by addition of rIL-1β (Fig. 4H). Although IL-25 mRNA could not be detected in these experiments, Sonobe *et al.* [43] have reported that IL-1β can down regulate IL-25 production by brain capillary endothelial cells indicating cross-talk between these cytokines and stromal cells. Together, these data indicate that IL-1β acts to negatively regulate helminth-induced IL-25 and IL-33 production, possibly through actions on IEC.

We next investigated the contribution of these cytokines to the expulsion of adult worms from the intestine. Interestingly, mice lacking IL-25 exhibited elevated worm burdens and fecal egg counts, whilst animals lacking the IL-33R (T1/ST2) were comparable to WT controls (Fig. 4I and Fig. S3C). Animals deficient in both the IL-25 and IL-33 receptors exhibited an intermediate phenotype indicating that IL-33 may actually promote worm chronicity. Taken together, these data indicate that IL-1β acts to suppress the production of Hp-elicited IL-25 and IL-33 and that the ability of IL-1β to attenuate IL-25 likely accounts for its impact on parasite chronicity.

### IL-1β limits the expansion of innate-like lymphoid cells (ILC)

IL-25 is a potent stimulator of a novel cell population commonly referred to as innate lymphoid cell type 2 (ILC2) [44]. These cells lack markers of known leukocyte lineages, but express c-kit, ICOS, TI/ST2 (IL-33R) and variable levels of Sca-1 [40], [45], [46], [47]. They represent a potent early source of IL-13 and IL-5 and promote goblet cell hyperplasia, eosinophil hematopoesis and adaptive type 2 responses [40], [45], [48]. *N. brasiliensis* infection is a potent inducer of ILC2 expansion, and although these cells are relatively rare in WT mice following Hp infection their numbers could be substantially increased upon additional treatment with exogenous rIL-25 [49].

**Figure 5:**
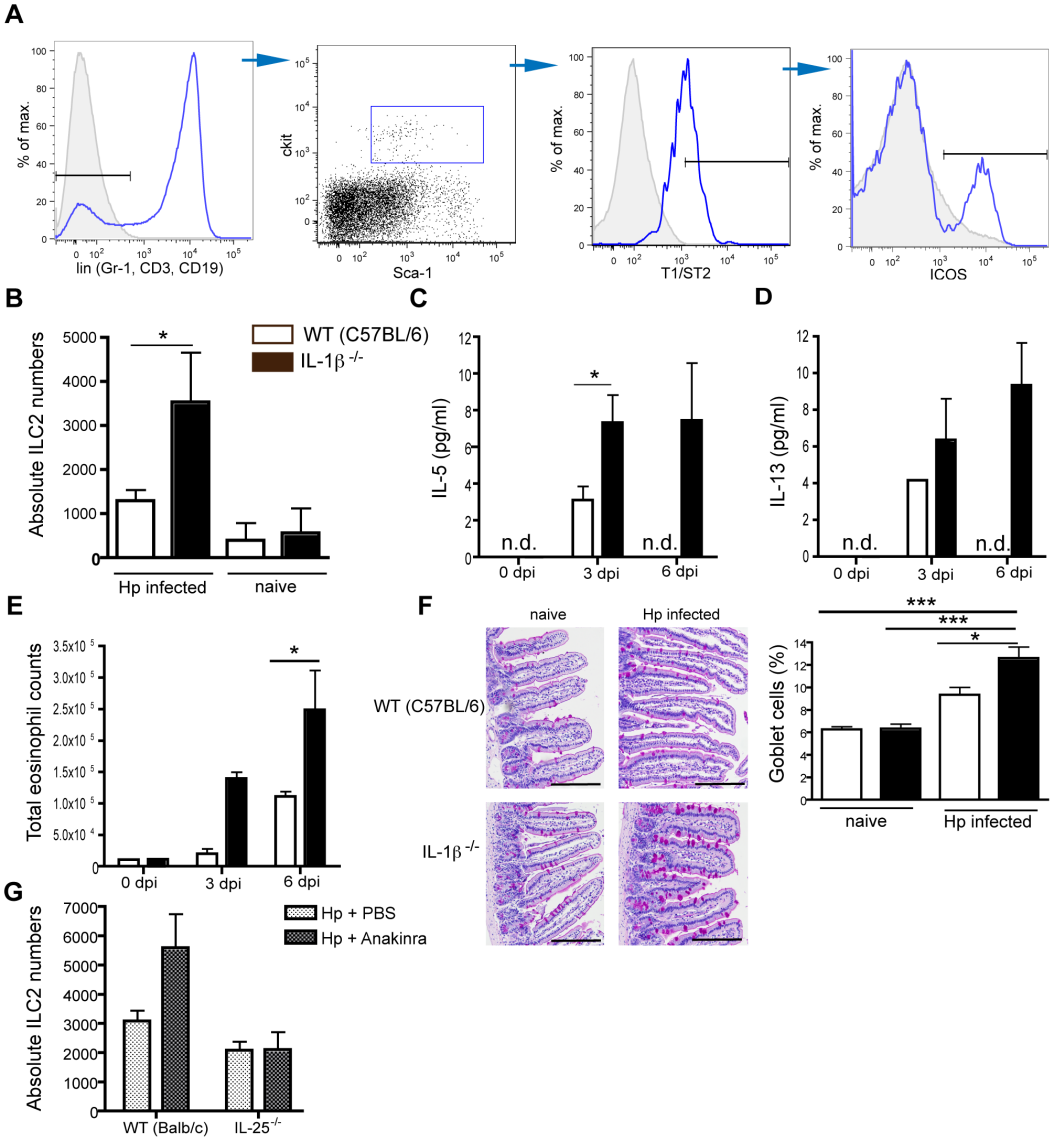
IL-1β attenuates Hp-induced increases in type 2 innate lymphoid cells (ILC2). WT (C57BL/6), WT (Balb/c), IL-1β^−/−^ and IL-17Br×T1ST2^−/−^ mice were administered 200 L3 by oral gavage. (**A**) ILC2 cells were identified as lineage-negative (Gr-1, CD3, CD19), Sca-1, c-kit, T1/ST2 and ICOS positive live cells present in the spleen of mice at 6 dpi. (**B**) Absolute numbers of splenic ILC2 cells and levels of (**C**) IL-5 and (**D**) IL-13 protein present in the peritoneal wash at 3 and 6 dpi as determined by ELISA. (**E**) Absolute numbers of eosinophils present in the peritoneal wash at the indicated timepoints following Hp infection were determined by differential cell counting of cytospins. (**F**) Intestinal sections were stained with PAS and the percentage of intestinal epithelial cells represented by PAS^+^ goblet cells determined. Scale bars represent 200 µm. (**G**) WT (Balb/c), IL-25^−/−^ and IL-17BR×T1ST2^−/−^ mice were treated daily with 100 µl of PBS or PBS plus 50 mg/kg Anakinra for 6 consecutive days starting at day 0 of Hp infection. Absolute numbers of ILC2 cells were determined in the spleen at 6 dpi. All data are representative of at least 2 independent experiments (n = 5–10 per group) and expressed as mean ± SEM.

We investigated the presence of ILC2s following Hp infection of WT or IL-1β^−/−^ mice. ILC2s were identified by staining various tissues for lineage negative (Gr-1, CD3, CD19), sca-1, c-kit, T1/ST2, ICOS positive cells (Fig. 5A). Hp infection elicited a small but non-significant increase in ILC2s in the spleen at 6 dpi (Fig. 5B). Importantly ILC2 expansion was markedly increased in IL-1β^−/−^ mice (Fig. 5B), and their identity as ILC2s was confirmed by an extended lineage negative panel (Fig. S4A) and by their potential to produce IL-13 (Fig. S4B). The increased ILC2 expansion in IL-1β^−/−^ mice correlated with increased early IL-5 and IL-13 cytokine production (Figs. 5C–D), helminth-induced eosinophilia (Fig. 5E) and goblet cell hyperplasia (Fig. 5F). The stronger increase in ILC2 numbers in the absence of IL-1β following Hp infection could be confirmed by pharmacological blockade with Anakinra (Fig. 5G), and Anakinra treated mice additionally exhibited increased eosinophilia and IL-5 and IL-13 cytokine levels (Figs. S4C–E). To determine whether IL-25 played a role in promoting Hp-induced ILC2 expansion in the absence of IL-1β we treated IL-25^−/−^ mice with Anakinra. As expected, Anakinra treatment of IL-25^−/−^ mice failed to expand ILC2 numbers (Fig. 5G), confirming an important role for this cytokine in Hp-induced ILC2 expansion.

### Increased ILC2 numbers alone are not sufficient for expulsion of Hp

**Figure 6:**
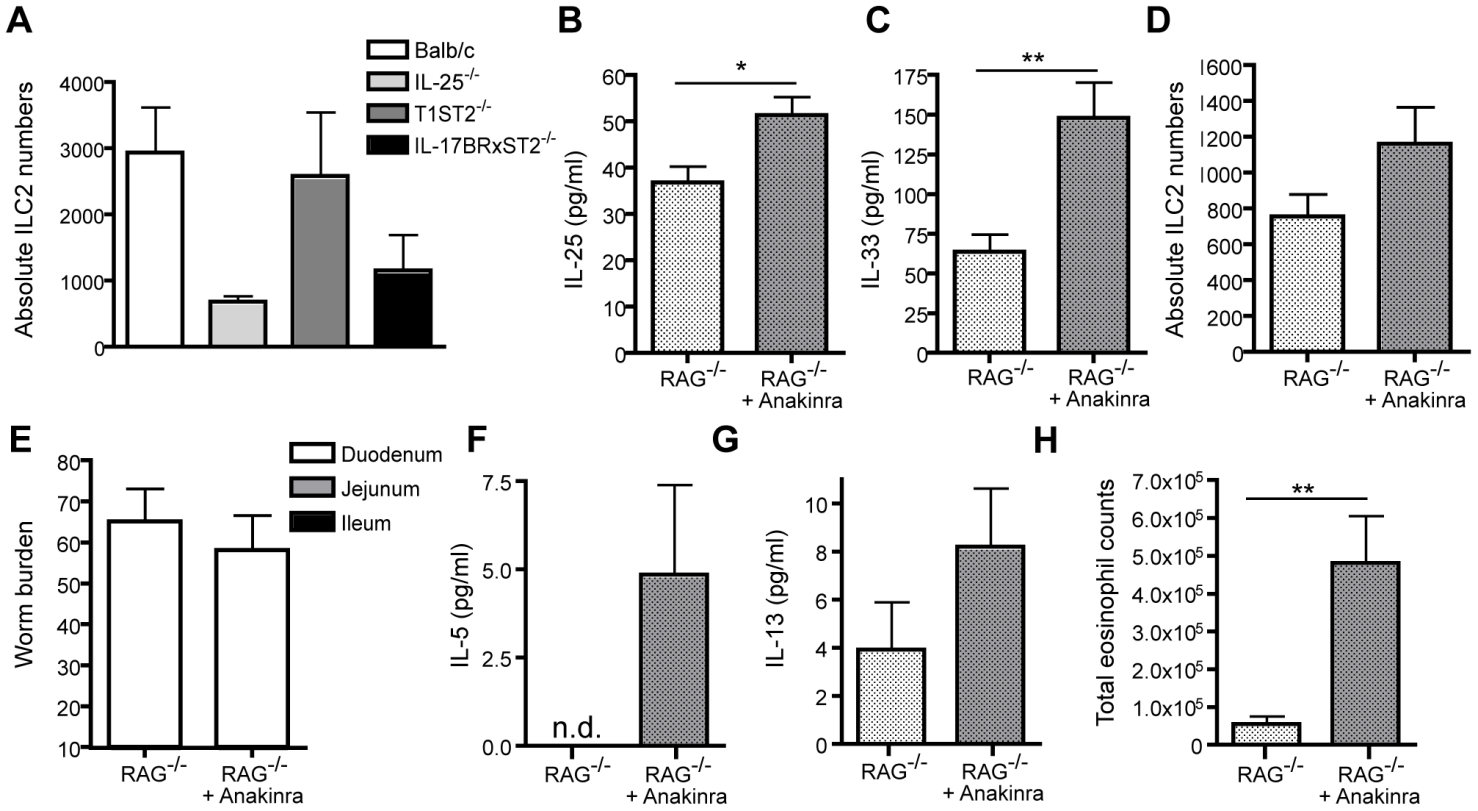
ILC2s alone are not sufficient enough to reject Hp worms and function mainly to support Th2 responses. WT (Balb/c), RAG^−/−^, IL-25^−/−^, T1ST2^−/−^ and IL-17Br×T1ST2^−/−^ mice were administered 200 L3 Hp by oral gavage. (**A**) ILC2 numbers in WT (Balb/c), IL-25^−/−^, T1ST2^−/−^ and IL-17Br×T1ST2^−/−^ mice were determined in the spleen at 6 dpi. Hp infected RAG^−/−^ mice additionally received 100 µl of PBS (RAG^−/−^) or PBS plus 50 mg/kg Anakinra (RAG^−/−^+Anakinra) via i.p. injection every day until 6 dpi to analyze, (**B**) IL-25 and (**C**) IL-33 cytokine levels in intestinal duodenum tissue cultures. (**D**) Absolute splenic ILC2 numbers. (**E**) Hp infected RAG^−/−^ mice additionally received 100 µl of PBS or PBS plus 50 mg/kg Anakinra via i.p. injection from 0–10 dpi and total worm numbers were analyzed at 13 dpi. (**F**) IL-5 and (**G**) IL-13 protein present in the peritoneal wash at 6 dpi as determined by ELISA. (**H**) Absolute numbers of eosinophils present in the peritoneal wash at 6 dpi were determined by differential cell counting of cytospins. All data are representative 3 independent experiments (n = 5–10 per group) and expressed as mean ± SEM.

Absolute ILC2 numbers in Hp infected WT (Balb/c), IL-25^−/−^, T1ST2^−/−^ and IL-17BR×T1ST2^−/−^ mice (Fig. 6A) showed a negative correlation to worm burdens (Fig. 4I), indicating a possible role in worm expulsion as reported for ILC2s in *N. brasiliensis* infection [40], [45], [46], [47], [48], [50], [51]. We therefore investigated whether increased ILC2 numbers in the absence of IL-1β^−/−^ were sufficient to promote Hp expulsion. RAG^−/−^ mice, which harbor ILC2s but lack CD4^+^ T cells, were treated with Anakinra following Hp infection resulting in increased duodenal IL-25 (Fig. 6B), IL-33 (Fig. 6C). These data correlated with a tendency towards increased splenic ILC2 numbers, although this did not reach statistical significance (Fig. 6D). However, in contrast to WT mice (Fig. 2H), Anakinra treatment of RAG^−/−^ mice failed to impact on total worm numbers or worm location within the intestinal lumen (Fig. 6E). RAG^−/−^ mice, either with or without Anakinra treatment, also failed to generate granulomas in response to Hp larval invasion (data not shown), despite exhibiting increased IL-5 (Fig. 6F) levels and increased peritoneal eosinophilia (Fig. 6H). As for ILC2 numbers, IL-13 levels reproducibly demonstrated a slight increase following Anakinra treatment, however this did not reach statistical significance (Fig. 6G). Taken together, these data indicate that early IL-5 production and peritoneal eosinophilia occur independently of T cells following Hp infection. As ILC2s are known to be present in RAG^−/−^ mice [45] our data also indicates that these cells are not sufficient to promote granuloma formation or parasite expulsion. However, our data does not rule out a role for ILC2s during Hp infection of T and B cell competent mice by promoting the proper expansion and differentiation of Th2 cells.

## Discussion

**Figure 7:**
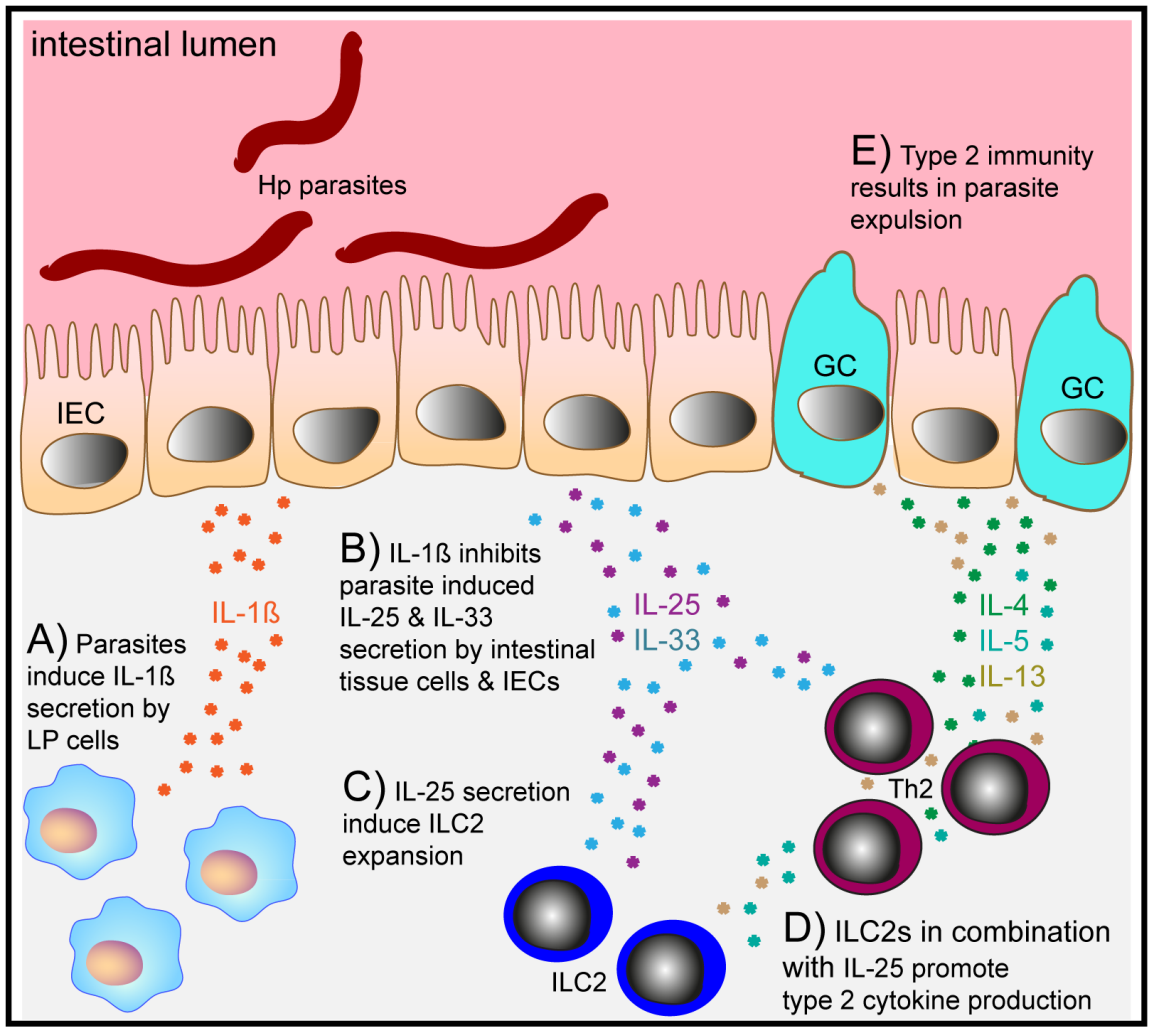
Hp induced IL-1β production promotes helminth chronicity. (**A**) Parasites induce IL-1β secretion by lamina propria (LP) cells. (**B**) IL-1β inhibits parasite induced IL-25 and IL-33 secretion by intestinal tissue cells and intestinal epithelial cells (IECs). (**C**) IL-25 secretion induce innate lymphoid cells type 2 (ILC2s). (**D**) ILC2s in combination with IL-25 promote type 2 cytokine production. (**E**) Type 2 immunity, including goblet cell hyperplasia and smooth muscle contraction result in expulsion of the adult Hp worms.

The potential of STH to evade host immunity is an essential part of their survival strategy. Infection with Hp, a natural parasite of murine rodents, normally elicits a Th2-type immune response that nevertheless fails to expel the parasite, resulting in chronic infection. In the present study we have identified a novel role for IL-1β in attenuating type 2 immunity and promoting parasite chronicity (Fig. 7). The role of IL-1β in modulating type 2 immunity has been addressed in prior studies with conflicting results. Early studies highlighted the requirement for IL-1 in the development of Th2 cells *in vitro* [52], [53], [54], whilst other studies showed an involvement of IL-1 in promoting IFNγ secretion by Th1 cells [55] and a suppressive effect of IL-1α and IL-1β on IL-4 secretion by human T cells [56]. *In vivo*, IL-1R1 deficiency was shown to result in enhanced Th2-type immune responses following infection with the protozoan parasite *Leishmania major* [57] and IL-1α was reported to promote Th1-biased immune resistance to *Leishmania major* [58]. In contrast to these findings, both IL-1α^−/−^ and IL-1β^−/−^ mice exhibit defective Th2-mediated resistance to the helminth *T. muris* [59], [60], and IL-1α, IL-1β have been reported to be necessary for promoting airway inflammation [61] and particle-induced pulmonary inflammation [62], respectively. Our data clearly shows a role for IL-1β in attenuating protective type 2 immunity following Hp infection, however it will be necessary to study the role of this cytokine in modulating resistance against other species in order to gain a thorough picture of its exact role during helminth infection.

Following Hp infection IL-1β was predominately expressed by hematopoietic cells – most likely intestinal and peritoneal macrophages. Hp products could induce both the expression of proIL-1β and inflammasome activation resulting in the cleavage and maturation of IL-1β, in an Nlrp3 and ASC-dependent manner. Although IL-1β is normally associated with protective immunity against pathogens, our data indicate a novel role for this cytokine in promoting pathogen chronicity. IL-1β gene deficiency, or the use of the IL-1R1 antagonist Anakinra, resulted in faster worm expulsion following Hp infection and reduced worm fecundity. These findings coincided with increased type 2 intestinal granuloma formation, ILC2 expansion and Th2 cytokine production. In turn, this response led to increased helminth-induced eosinophilia, goblet cell hyperplasia and serum IgE. Interestingly, restricting IL-1β blockade to the first 10 days of infection also resulted in a reversal of parasite chronicity indicating that the strength of the type 2 immune response during the acute phase of infection can result in parasite damage that impacts on the ability of the worm to establish chronicity. The early burst of IL-1β production observed following Hp infection would thus be expected to be adequate to allow the parasite to escape such damage and to establish chronicity.

In our study IL-1β was additionally determined to suppress helminth-induced IL-25 and IL-33. IECs are thought to represent the major source of early IL-25 and IL-33 following helminth infection [38], [48], [63]. In keeping with this, we identified IECs within the intestinal tissue as a dominant source of IL-25 and IL-33 production following Hp infection. We believe that IL-1β may act directly on IECs to attenuate IL-25 production as we and others have shown that IECs express the IL-1R1 [64], [65], [66]. We could also demonstrate IL-1β-mediated inhibition of HES-induced IL-33 mRNA by murine IEC *in vitro*. However IL-25 and IL-33 can also be produced by other cell types, such as macrophages and mast cells [41], [67], and it is possible that IL-1β additionally modulates cytokine levels by acting on these cells. Determination of the exact molecular mechanisms by which IL-1β can attenuate IL-25 and IL-33 expression will be important for future studies.

IL-25 and IL-33 have recently been identified as potent inducers of type 2 immunity through their actions on ILC2 [47], [48] and CD4^+^ T cells [37], [68]. We noted that IL-25 and IL-33 production in WT mice was relatively poor following Hp infection, perhaps explaining why this parasite establishes chronicity, whilst *N. brasiliensis*, which elicits a strong IL-25 and IL-33 response [46], [63], [69], does not. Hepworth *et al.*, [49] also reported negligible increases in ILC2 numbers in WT mice after Hp infection and we could reproduce these data. Interestingly, IL-1β^−/−^ mice exhibited increased ILC2 numbers, a finding likely explained by the impact of IL-1β on IL-25 as Hepworth *et al.*, [49] could show that rIL-25 treatment of Hp infected mice expanded ILC2s, and we showed that ILC2 expansion following Anakinra treatment was IL-25 dependent. IL-25 deficient mice exhibited increased worm burdens at late time-points post-infection, indicating that even in the presence of normal IL-1β signaling low levels of IL-25 have a critical role in mediating worm expulsion. Thus we speculate that the main function of IL-1β is to suppress exaggerated IL-25-induced immune responses that give rise to almost complete worm expulsion. This hypothesis is supported by a recent study showing that treatment of Hp infected WT mice with rIL-25 results in the acute expulsion of adult worm burdens [41].

Although we observed that IL-25 was required for Hp-induced ILC2 expansion, RAG^−/−^ mice which retain ILC2s but lack CD4^+^ T cells, did not form granulomas and failed to expel parasites following Hp infection. These data indicate that Th2 cells are required for parasite expulsion. Previous studies indicate that ILC2s can act to promote tissue eosinophilia, goblet cell hyperplasia and expansion of Th2 cells [40], [45], [46], [47], [50]. IL-25 can also act on a number of cell types to promote type 2 immunity including ILC2s [47], antigen-presenting cells [70], invariant NKT cells [71], mast cells [72] and Th2 cells [37], [73]. Thus, increased IL-25 production in the absence of IL-1β may act to promote Hp expulsion by Th2 cells in a direct manner, or indirectly through its effects on other cell subsets.

In conclusion, our data reveals a novel role for IL-1β in promoting helminth chronicity. The means by which IL-1β achieves this includes, but may not be limited to, i) suppression of helminth-elicited IL-25 and IL-33 secretion, ii) diminished ILC2 numbers and iii) attenuated Th2-type immune responses (Fig. 7). This course of events allows Hp to escape Th2-dependent immune damage and to establish a chronic infection. Of note, IL-25 and IL-33 have been reported to modulate the severity of various inflammatory diseases involving IL-1β secretion including allergic inflammation, autoimmunity and inflammatory bowel disease [74], [75]. Thus, our findings are likely to have important implications for numerous diseases in addition to helminth infection.

## Materials and Methods

### Ethics statement

All animal experiments were approved by the office Affaires vétérinaires (1066 Epalinges, Canton Vaud, Switzerland) with the authorization Number 2238 according to the guidelines set by the Service de la consommation et des affaires vétérinaires federal (Canton Vaud, Switzerland).

### Mice, parasites and treatments

C57BL/6, Balb/c, Asc^−/−^ [76], Nlrp3^−/−^ [76], Trif^−/−^ [77], MyD88^−/−^ [78], IL-1β^−/−^ [79], IL-1R1^−/−^ [80], RAG-1^−/−^ [81], IL-25^−/−^ [47], T1/ST2^−/−^ [82] and IL-17BR×T1ST2^−/−^ [45] mice were bred and maintained under specific pathogen-free (SPF) conditions at Ecole Polytechnique Fédérale de Lausanne (EPFL) or Centre Hospitalier Universitaire Vaudois (CHUV) at Epalinges, Switzerland. To standardize the intestinal bacteria within different groups of mice contained within one experiment, all mice were co-housed or beddings were mixed for 2–3 weeks prior to parasite infection. Where indicated mice were then infected orally with 200 L3 Hp. For pharmacological blockade of IL-1β, mice were additionally treated with 0.1 ml PBS or 50 mg/kg Anakinra in 0.1 ml PBS via i.p injection once daily, starting on day 0 of the infection. Adult worm burdens and granuloma numbers were determined by manual counting using a dissecting microscope. Egg production was quantified by collection of moist feces, flotation using saturated NaCl, and counting using a McMaster Worm Egg Counting Chamber (Weber Scientific International, Ltd, Hamilton, NJ, USA).

### Generation and collection of HES products

For the generation of Hp excretory/secretory (HES) products, L5 Hp helminths were washed extensively in sterile PBS supplemented with penicillin and streptomycin (Gibco), then incubated for 1 h in RPMI (Gibco) supplemented with penicillin and streptomycin and cultured in RPMI plus antibiotics (penicillin, streptomycin, and gentamicin; Sigma–Aldrich) and 1% glucose (Sigma–Aldrich). The supernatant was collected every 2 days for a period of 2 weeks, followed by sterile filtration and concentration of the supernatant by centrifugation through a 10,000 MWCO cellulose membrane (Centriprep; Millipore). LPS contamination was removed from HES using an EndoTrap Blue LPS-binding affinity column (Hyglos GmbH, Germany). The concentration of residual endotoxin was determined using the Limulus Assay, which has a sensitivity of 0.06 Endotoxin Units/ml (6 pg/ml) (Lonza). The final preparation used for this study contained 31 pg/ml LPS in the pyrogen-free HES (P.HES) vs. 643 pg/ml in the non-purified HES (HES).

### Bone marrow chimeras

For the generation of bone marrow (BM) chimeras, C57BL/6 and IL-1β^−/−^ mice were lethally irradiated (9.5 Gy) using a 60 Cobalt source then injected intravenously with 5×10^5^ BM cells depleted of CD4^+^ T cells by MACs separation. BM chimera mice were then treated with Biafine crème for 3 days and with Bactrim (60 mg/kg/day) and Paracetamol (200 mg/kg) for 14 days in the drinking water. 21 days after BM transfer mice were infected with Hp.

### Mononuclear cell isolation from intestinal lamina propria tissues

Small intestinal lamina propria cells were isolated as previously described [83]. Briefly, the colon was flushed, opened longitudinally, washed thoroughly in Mg_2_Cl_2_- and CaCl_2_-free DPBS (GIBCO, Invitrogen, Gaithersburg, MD). The tissues were then cut into 3–5 mm pieces that were incubated 4–5 times in 25 ml EDTA/HEPES/DPBS solution at 37°C for 20 min in a shaking incubator in order to remove the epithelial layer. Intestinal pieces were collagenase-digested for 40 min at 37°C in 25 ml IMDM containing 0.5 mg/ml collagenase type VIII (Sigma-Aldrich, Taufkirchen, Germany), 50 U DNaseI (Roche Diagnostic, Nutley, NJ), and 0.01 M HEPES (GIBCO, Invitrogen). The crude cell suspension was loaded onto a 30%/100% percoll (GE Healthcare, Milan, Italy) gradient and centrifuged at 680 g for 30 min at room temperature with the acceleration and brake turned off. Cells were collected from the 30%/100% interphase and further used for CD11b positive cell isolation using MACS cell separation technique (Miltenyi Biotech) according to the manufacturer's instructions.

### Identification of helminth-induced type 2 CD4^+^ T cells and ILC2s

Hp induced cytokine production by T cells was assessed by culturing cell suspensions from the mesenteric lymph node (MLN) or spleen 72 h in the presence of 5 µg/mL HES at 37°C. At the end of this period cells were additionally stimulated 4 h with PMA and ionomycin, with brefeldin A added for the last 2 h of culture. Cells were then harvested and stained for surface markers, then fixed with 2% paraformaldehyde before staining with anti-cytokine antibodies. Antibodies against mouse CD3, CD4, CD11b, CD11c, CD19, Nk1.1, CD19, CD45, CD127, GR-1, c-kit, IL-4, IL-7Rα and IgE were from BioLegend; IL-13, Sca-1 from eBioscience and T1/ST2 from Mdbioproducts. For detection of ILC2s spleen cell suspensions were stained with biotinylated CD3, CD4, CD11b, CD11c, CD19, Nk1.1, Gr-1 and IgE or CD3, CD19 and Gr-1, followed by streptavidin labeled Texas Red, Sca-1 (PE-Cy7), c-kit (Pacific Blue), T1/ST2 (FITC), and ICOS (PerCP/Cy5.5) and subjected to a gating strategy as shown in Figure 7A. All flow cytometry analyses were performed on a LSRII (BD) and analyzed using FlowJo software (TreeStar).

### Peritoneal wash and intestinal tissue culture

Peritoneal washes were performed with 0.5 ml cold PBS using 2–3 washes. For intestinal tissue culture the intestine was removed, extensively flushed with cold PBS, divided into duodenum, jejunum and ileum, cut longitudinally and placed separately in 100 mm Petri dishes with PBS containing 10 µg/ml Gentamicin, 100 U/ml Penicillin and 100 µg/ml Streptomycin (all from GIBCO). Opened cut intestines were gently scraped to remove mucus, washed 3 times and transferred into a new Petri dish. From each part of the intestine (duodenum, jejunum and ileum) the anterior 4 cm were cut into 1–2 mm pieces and transferred to a 24-well plate with 2 ml/well of RPMI media (PAA Laboratories) containing 10% heat inactivated FCS, 10 µg/ml Gentamicin, 100 U/ml Penicillin and 100 µg/ml Streptomycin (all from GIBCO). 24-well plates were incubated over night at 37°C before supernatants were harvested and analyzed by ELISA. Cytokine concentrations were normalized against tissue weight and are presented as pg/mg tissue.

### ELISA and Milliplex

Serum samples were analyzed by mouse Milliplex Kit (Merk Millipore) for IL-4. Peritoneal wash or intestinal tissue culture supernatants were analyzed by mouse ELISA for IL-1β, IL-5, IL-33 (eBiosciences) or IL-25 (BioLegend) according to the manufacturer's instructions. ELISA assays for total IgE and IgG were performed as previously described [12]. Antigen-specific IgG1, IgG2a and IgG2b were measured in a similar manner after coating with 1–5 mg/ml HES excretory/secretary products collected from adult L5 Hp cultured for a period of 2 days in RPMI plus antibiotics and 1% glucose and concentrated using a 10,000 MWCO cellulose membrane, Centriprep, Millipore, MA). To calculate concentrations an internal standard consisting of pooled serum from C57BL/6 or mice infected two times with Hp was used.

### Real-Time PCR

Tissues were stored in RnaLater (Ambion) or directly transferred to TRIzol (Invitrogen). RNA was extracted according to the manufacturer's instructions. Gene expression results are expressed as arbitrary units relative to expression of the house keeping gene β-actin. Primer sequences are as follows: IL-25 (5′-CAG CAA AGA GCA AGA ACC-3′ and 5′-CCC TGT CCA ACT CAT AGC-3′), IL-33 (5′- CAATCAGGCGACGGTGTGGATGG-3′ and 5′- TCCGGAGGCGAGACGTCACC-3′) and ß-actin (5′-CTT TTC ACG GTT GGC CTT AG-3′ and 5′-CCC TGA AGT ACC CCA TTG AAC-3′).

### In vitro inflammasome assay and Western blot

Murine bone marrow derived macrophages (BMM) were isolated and cultured as previously described [84], [85]. The day before stimulation, cells were collected and plated into flat-bottom 96-well tissue culture plates at 0.2×10^6^ cells per well. Where indicated, cells were primed with 20 ng/mL ultrapure *E.coli* K12 LPS (Invivogen) for 3 hrs followed by MSU (300 µg/mL) for an inflammasome positive control. Stimulation with HES (5 or 50 µg/mL), pyrogen-free HES (P.HES) (5 or 50 µg/mL), homogenized *H. polygyrus* L5 parasite (HPL5) (100 µg/mL) were performed for the indicated times. Cell supernatants were removed and kept for ELISA or Western blot. The cell pellet was washed with PBS then re-suspended directly in SDS sample buffer. For Western blot analysis of cell extracts, triplicate samples were pooled, incubated at 95°C for 5 minutes then separated on 15% SDS-page gels, transferred onto nitrocellulose membranes and blotted with polyclonal antibodies against mouse IL-1β (sheep) and caspase-1 (rabbit) which were generous gifts from R. Solari (Glaxo) and P. Vandenabeele (Ghent University, Belgium), respectively. Band intensity for IL-1β and β-actin was determined using Adobe Photoshop CS3. For band intensity normalization graph of IL-1β in CD11b^+^ lamina propria cells, each sample is normalized to naïve CD11b^−^, set as 1, for both IL-1β and β-actin. The ratio between IL-1β and β-actin band intensity was then determined. IL-1β is expressed as percent of β-actin intensity.

### Isolation of IECs

IECs were isolated as described previously [63]. Intestines were cut into 2–3 mm fragments and washed by PBS five times with vigorous shaking before incubation with 3 mM EDTA and 0.5 mM dithiothreitol at room temperature for 30 min with shaking. Epithelial cells released from the intestine by shaking were washed with PBS and transferred into TRIzol. To test for contamination of leukocytes, washed epithelial cell suspensions were digested by collagenase IV, and single cells were stained by CD45 antibodies. Only 3.01%±0.45 in WT and 3.27%±0.57 in IL-1β^−/−^ of the acquired cells were stained positive for CD45.

### MSIE culture

MSIE cell line [42] cells were expanded at 33°C with 5% CO_2_ in the presence of IFNγ. Cells were further differentiated for 2 days at 37°C without IFNγ after which they were stimulated for 2 h (for RNA extraction) or 24 h (for supernatant collection and protein analysis) with papain (Calbiochem) or HES (L5 Hp excretory secretory products) alone, or in combination with different concentrations of rIL-1β (R&D systems).

### Histology and goblet cell measurements

4 µm paraffin sections of swiss rolls from different sections of the small intestine were stained with PAS for evaluation of goblet cells. Goblet cell hyperplasia was determined by counting the number of PAS positive cells in 10 villi per section. The results were expressed as percentages of PAS positive cells per total epithelial cells. For expression of IL-1R1, slides were pretreated with heat induced epitope retrieval (HIER) before incubation over night at 4°C with the primary goat anti-mouse IL-1R1 antibody (R&D Systems, Minneapolis, MN, USA), followed by a secondary anti-goat ImmPRESS antibody for 30 min at RT. Histological slides were analyzed under the microscope (Olympus AX70), pictures were taken with the Olympus DP70 camera using DPController (Olympus) acquisition software.

### Statistical analysis

Statistical analysis was performed using a Student's t test, one-way or two-way ANOVA with post test as appropriate. All experiments were conducted independently at least twice P-values of <0.05 were considered significant and are shown as p<0.05 (*), p<0.01 (**), or p<0.001 (***). Graph generation and statistical analyses were performed using Prism version 4c software (GraphPad, La Jolla, CA).

## Supporting Information

Figure S1 THP-1 cells were transfected with shRNA for ASC, NLRP3 or mock. After 3 hours of PMA stimulation cells were activated with HES (5 µg/mL), P.HES (5 µg/mL) or HPL5 (5 µg/mL) for 18 hours. Cell supernatants were used for ELISA detection of IL-1β.
(TIF)

Figure S2 Mice were administered 200 L3 Hp by oral gavage. At 13 dpi single cell suspensions were made from (**A**) spleen, (**B**) MLN and cells were restimulated with HES as described in the Materials and Methods. The percentage of CD4^+^ T cells secreting IL-17 in WT (C57BL/6) are shown. At 13 dpi absolute cell numbers of CD4^+^ CD25^+^ foxp3^+^ T cells in (**C**) spleen and (**D**) MLN are shown for WT (C57BL/6) vs. IL-1β^−/−^ mice. All results are representative of at least 1 experiment (n = 6 per group) and expressed as mean ± SEM.
(TIF)

Figure S3 Mice were administered 200 L3 Hp by oral gavage. WT (C57BL/6) additionally received 100 µl of PBS or PBS plus 50 mg/kg Anakinra via i.p. injection every day and intestinal duodenum tissue cultures were analysed by ELISA for (**A**) IL-25 and (**B**) IL-33 cytokine levels at the indicated timepoints following Hp infection. (**C**) Fecal egg counts were determined for WT (Balb/c), IL-25^−/−^, T1/ST2^−/−^ and IL-17BR×T1ST2^−/−^ mice throughout the course of the experiment (data represents pooled feces of 2–3 individual cages per strain, n = 2–5 mice per cage).
(TIF)

Figure S4 Mice were administered 200 L3 Hp by oral gavage. (**A**) FACS analysis for absolute ILC2 numbers at 6 dpi with expanded straining for the lineage negative panel to CD3, CD4, CD11b, CD11c, CD19, Nk1.1, Ly6G and IgE. (**B**) FACS analysis for absolute ILC2 numbers at 6 dpi gated on IL-13 cytokine secreting ILC2s with the same lineage negative panel as in Fig. S4A. Mice were administered 200 L3 Hp by oral gavage. WT (C57BL/6) additionally received 100 µl of PBS or PBS plus 50 mg/kg Anakinra via i.p. injection every day and (**C**) numbers of eosinophils present in the peritoneal wash at 6 dpi were determined by differential cell counting of cytospins, (**D**) IL-5 and (**E**) IL-13 cytoine levels in the peritoneal wash at the 6 dpi were deterimend by ELISA.
(TIF)
