# Supplementary figures and images for: IL-1β Suppresses Innate IL-25 and IL-33 Production and Maintains Helminth Chronicity
Source: PLoS Pathog. 2013 Aug 1;9(8):e1003531. doi: 10.1371/journal.ppat.1003531 (PMC3731249; doi:10.1371/journal.ppat.1003531)

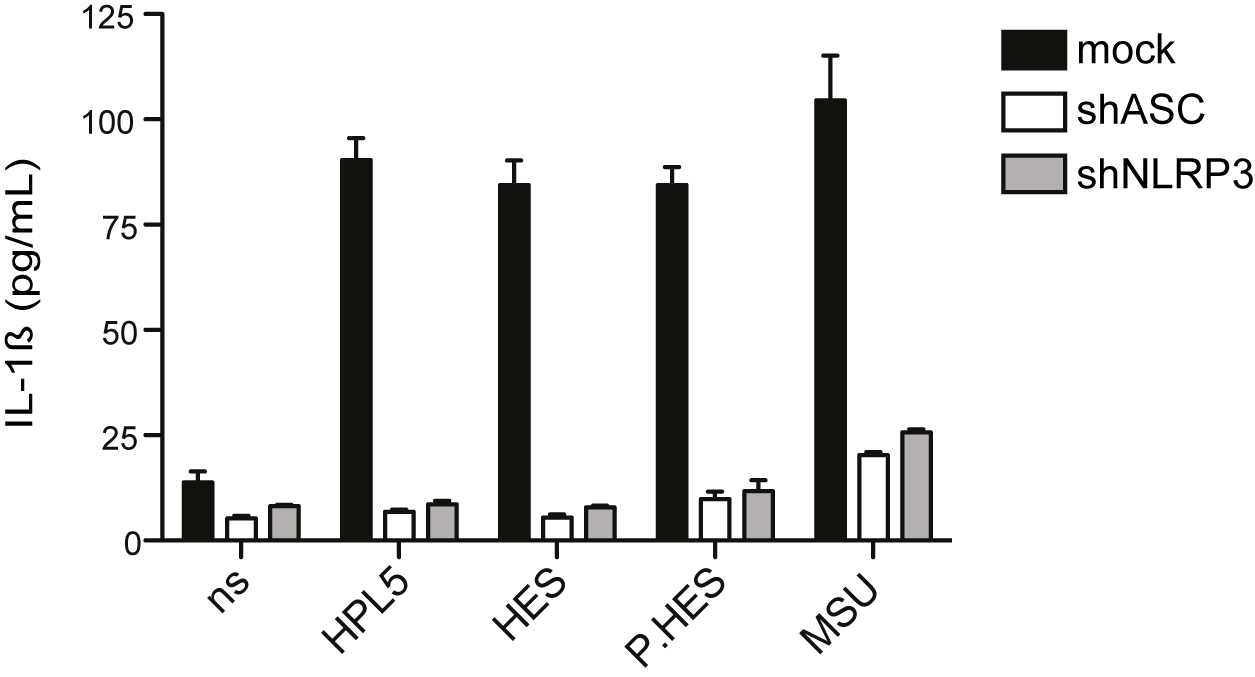

Supplement: Figure S1 — THP-1 cells were transfected with shRNA for ASC, NLRP3 or mock. After 3 hours of PMA stimulation cells were activated with HES (5 µg/mL), P.HES (5 µg/mL) or HPL5 (5 µg/mL) for 18 hours. Cell supernatants were used for ELISA detection of IL-1β. (TIF) [file ppat.1003531.s001.tif]

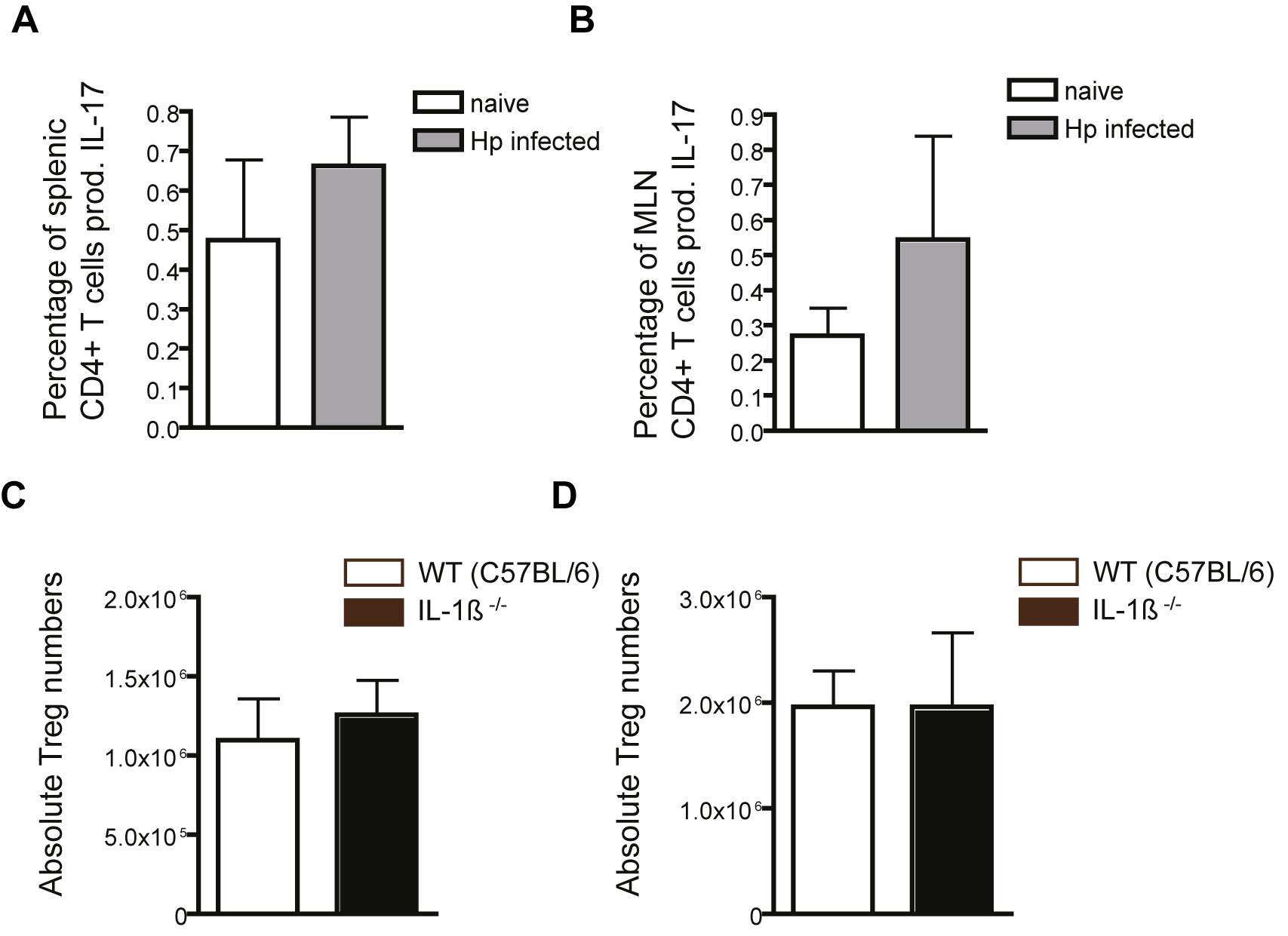

Supplement: Figure S2 — Mice were administered 200 L3 Hp by oral gavage. At 13 dpi single cell suspensions were made from (A) spleen, (B) MLN and cells were restimulated with HES as described in the Materials and Methods. The percentage of CD4+ T cells secreting IL-17 in WT (C57BL/6) are shown. At 13 dpi absolute cell numbers of CD4+ CD25+ foxp3+ T cells in (C) spleen and (D) MLN are shown for WT (C57BL/6) vs. IL-1β−/− mice. All results are representative of at least 1 experiment (n = 6 per group) and expressed as mean ± SEM. (TIF) [file ppat.1003531.s002.tif]

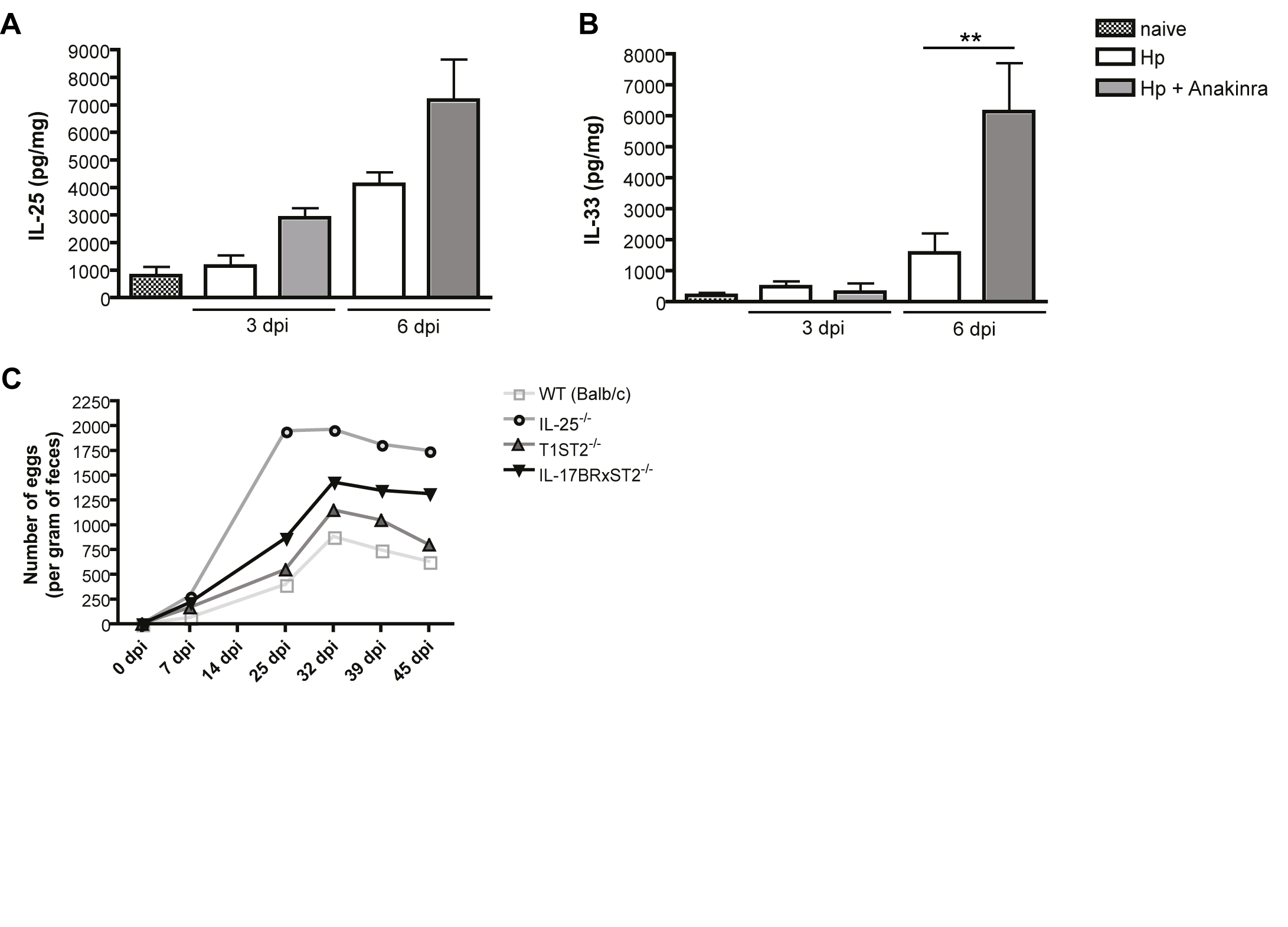

Supplement: Figure S3 — Mice were administered 200 L3 Hp by oral gavage. WT (C57BL/6) additionally received 100 µl of PBS or PBS plus 50 mg/kg Anakinra via i.p. injection every day and intestinal duodenum tissue cultures were analysed by ELISA for (A) IL-25 and (B) IL-33 cytokine levels at the indicated timepoints following Hp infection. (C) Fecal egg counts were determined for WT (Balb/c), IL-25−/−, T1/ST2−/− and IL-17BR×T1ST2−/− mice throughout the course of the experiment (data represents pooled feces of 2–3 individual cages per strain, n = 2–5 mice per cage). (TIF) [file ppat.1003531.s003.tif]

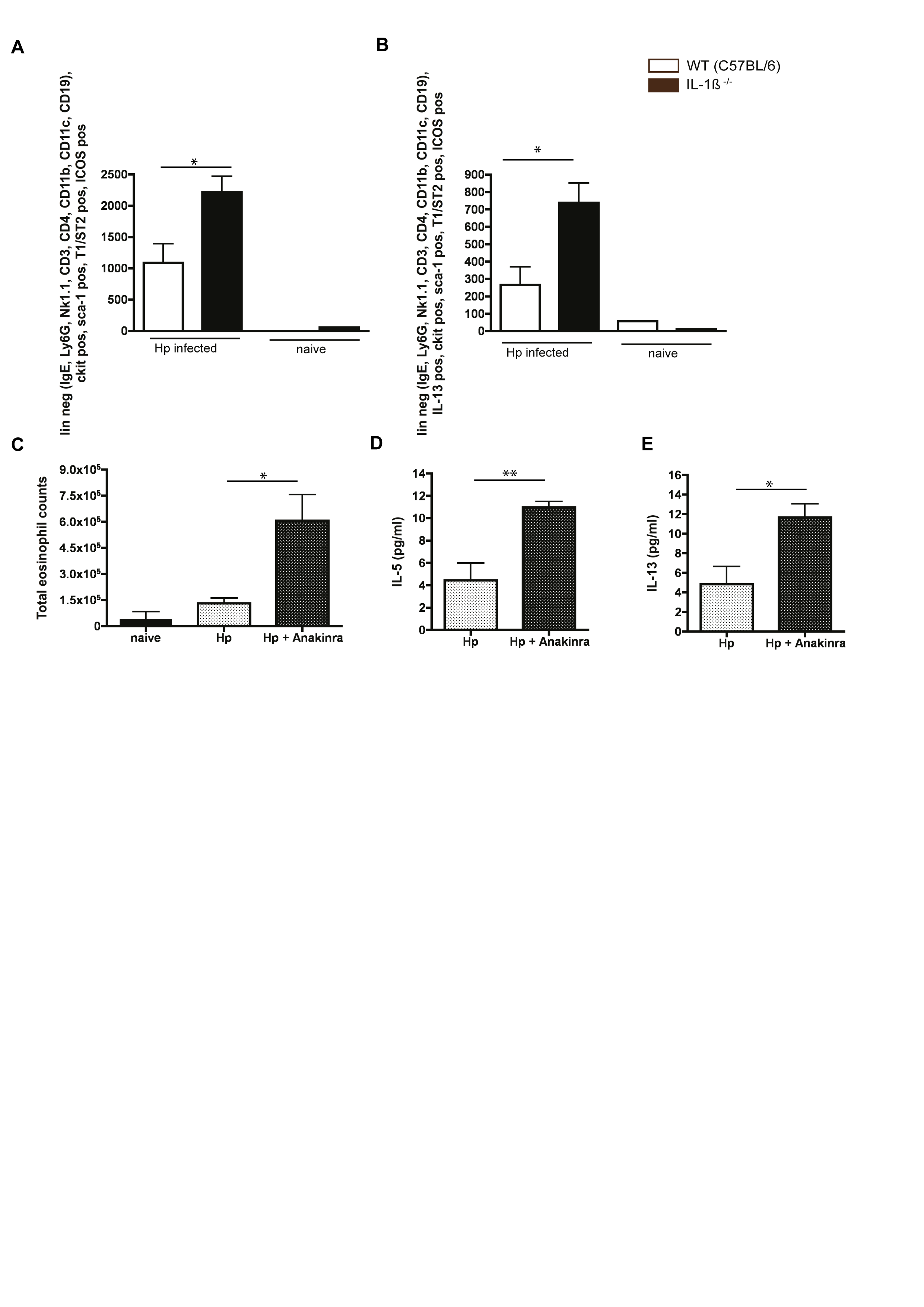

Supplement: Figure S4 — Mice were administered 200 L3 Hp by oral gavage. (A) FACS analysis for absolute ILC2 numbers at 6 dpi with expanded straining for the lineage negative panel to CD3, CD4, CD11b, CD11c, CD19, Nk1.1, Ly6G and IgE. (B) FACS analysis for absolute ILC2 numbers at 6 dpi gated on IL-13 cytokine secreting ILC2s with the same lineage negative panel as in Fig. S4A. Mice were administered 200 L3 Hp by oral gavage. WT (C57BL/6) additionally received 100 µl of PBS or PBS plus 50 mg/kg Anakinra via i.p. injection every day and (C) numbers of eosinophils present in the peritoneal wash at 6 dpi were determined by differential cell counting of cytospins, (D) IL-5 and (E) IL-13 cytoine levels in the peritoneal wash at the 6 dpi were deterimend by ELISA. (TIF) [file ppat.1003531.s004.tif]
